# Percutaneous intraductal radiofrequency ablation in the management of unresectable Bismuth types III and IV hilar cholangiocarcinoma

**DOI:** 10.18632/oncotarget.10116

**Published:** 2016-06-17

**Authors:** Yu Wang, Wei Cui, Wenzhe Fan, Yingqiang Zhang, Wang Yao, Kunbo Huang, Jiaping Li

**Affiliations:** ^1^ Department of Interventional Oncology, the First Affiliated Hospital, Sun Yat-sen University, Guangzhou 510080, China

**Keywords:** radiofrequency ablation, percutaneous transhepatic cholangial drainage, metal stent, hilar cholangiocarcinoma

## Abstract

**Purpose:**

To assess the feasibility and safety of percutaneous intraductal radiofrequency ablation (RFA) for unresectable Bismuth types III and IV hilar cholangiocarcinoma.

**Results:**

Percutaneous intraductal RFA combined with metal stent placement was successful in all patients without any technical problems; the technical success rate was 100%. Chemotherapy was administered to two patients. After treatment, serum direct bilirubin levels were notably decreased. Six patients died during the follow-up period. Median stent patency from the time of the first RFA and survival from the time of diagnosis were 100 days (95% confidence interval (CI), 85–115 days) and 5.3 months (95% CI, 2.5–8.1 months), respectively. No acute pancreatitis, bile duct bleeding and perforation, bile leakage, or other severe complications occurred. Four cases of procedure-related cholangitis, three cases of postoperative abdominal pain, and five cases of asymptomatic transient increase in serum amylase were observed. One patient who presented with stent blockage 252 days' post-procedure underwent repeat ablation.

**Materials and Methods:**

Between September 2013 and May 2015, nine patients with unresectable Bismuth types III and IV hilar cholangiocarcinoma who were treated with percutaneous intraductal RFA combined with metal stent placement after the percutaneous transhepatic cholangial drainage were included in the retrospective analysis. Procedure-related complications, stent patency, and survival after treatment were investigated.

**Conclusion:**

Percutaneous intraductal RFA combined with metal stent placement is a technically safe and feasible therapeutic option for the palliative treatment of unresectable Bismuth types III and IV hilar cholangiocarcinoma. Its long-term efficacy and safety is promising, but needs further study via randomized and prospective trials that include a greater number of patients.

## BACKGROUND

Hilar cholangiocarcinoma (Hi CC), also called perihilar cholangiocarcinoma or Klatskin tumor, is a common malignant tumor of the biliary tree with about 40%-60% of all cholangiocarcinoma cases [1, 2]. The tumor may arise from the primary biliary confluence, right or left hepatic ducts, secondary biliary confluence, or distal second order bile ducts (which together form the hilum) [3]. Its poor prognosis is closely related to its staging and regional invasion. Radical surgical resection is the only curative treatment, conferring a 5-year survival rate in the range of 20% to 42% [4]. Because of the nonspecific early stage symptoms, Hi CC is usually diagnosed only in the advanced stages, and only one-third of Hi CC patients undergo curative resection [5]. Hi CC is classified into four types according to Bismuth's scheme on the basis of the involvement of the hepatic confluence [6]. The patients with types I and II tumors had a high 5-year survival rate after R0 resection [7–9]. Although symptomatic patients are eligible for curative resection, hilar bile duct resection with major liver resection is the standard treatment for Bismuth types III and IV Hi CC [10], which confers a very poor surgical prognosis, often with further complications [11, 12]. For patients with Bismuth types III and IV Hi CC who cannot tolerate or benefit from surgery, the use of percutaneous transhepatic cholangial drainage (PTCD), chemotherapy, radiotherapy, or chemoradiation therapy as palliative treatment can alleviate obstructive symptoms, improving the quality of life and disease-free survival [13–15]. However, biliary stenting, chemotherapy, and radiotherapy only relieve symptoms in the short-term, and do not benefit intraductal tumor recurrence. Thus, it is extremely important to find a safe and efficient method to treat types III and IV Hi CC.

Radiofrequency ablation (RFA) has been widely recognized in the treatment of solid tumors, especially hepatocellular carcinoma. It has similar survival rate as liver resection and transplantation [16–18]. However, there are few studies about the clinical applicability of RFA for malignant bile duct obstruction. Recently, intraductal RFA has shown to confer satisfactory therapeutic effects. Additionally, the safety and efficacy of RFA catheter deployment combined stent placement has been demonstrated in patients with unresectable malignant obstructive jaundice [14, 19–21]. The RFA procedure can be performed either through an endoscopic or percutaneous route with the use of a special RFA catheter (Habib™ EndoHPB, EMcision Ltd, London, UK). In this study, we performed clinical study to select a percutaneous approach for RFA combined with non-covered self-expanding metal stent placement (SEMS) (WallFlex, Boston Scientific, Natick, MA) because RFA can be applied following PTCD with minimal discomfort to the patient, and metal stent placement is considered to relieve obstructive symptoms and improve quality of life [13]. Moreover, intraductal RFA may decrease tumor ingrowth and benign epithelial hyperplasia, achieve local tumor control, and prolong stent patency due to substantially increasing the diameter of malignant biliary strictures [22]. The purpose of this study was to evaluate the safety and efficacy of this procedure as well as the survival of patients with unresectable Bismuth types III and IV Hi CC.

## RESULTS

Between September 2013 and May 2015, 10 RFA combined with SEMS placement treatment cycles were performed in nine patients (seven men and two women) with a mean age of 65.3 ± 3.6 years (range, 46–77 years). All patients were diagnosed with unresectable Hi CC (Bismuth classification: Type IIIA, 1; Type IIIB, 1; Type IV, 7) . The median performance status was 1 with a range of 0–2 (Table 1).

**Table 1:** Patient characteristics

No.	Gender	Age	PS	Bismuth types	No. of RFA treatment cycles	Follow-up (mo.)	Outcome
1	M	75	1	IV	2	12.2	Alive
2	M	65	0	IIIB	1	4.5	Alive
3	M	69	1	IV	1	3.9	Dead
4	M	61	2	IV	1	5.3	Dead
5	F	77	2	IV	1	3.5	Dead
6	M	67	1	IV	1	4.0	Alive
7	M	52	2	IIIA	1	3.7	Dead
8	F	46	1	IV	1	3.2	Dead
9	M	76	2	IV	1	9.9	Dead

F: Female; M: Male; No. of RFA treatment cycles: the number of treatment cycles during follow-up.

Percutaneous RFA with SEMS placement was achieved successfully in all nine patients. The median energy and time used during single procedure was 10 W for 2 × 90 s. Most patients (8 of 9) underwent only one RFA session; re-RFA was only performed non-electively in one patient (No. 1; Table 1) because of a stent occlusion at 252 days post-procedure. The mean (± standard deviation [SD]) length of the biliary stricture was 5.18 ± 1.37 cm, and the median duration of each ablation was 90 seconds (range, 60–120 seconds). The ablation energy used was 10 Watts (8 of 9 patients; Table 2). The mean (± standard deviation [SD]) post-ablation direct bilirubin was 62.06 ± 17.21 umol/L reduced from a mean pre-ablation level of 87.88 ± 18.99 umol/L. The mean amylase on the first postoperative day was 97.67 ± 23.42 U/L reduced from a mean (± SD) postoperative quick-check level of 163.37 (± 47.36) U/L. The remaining routine blood and biochemical parameters remained largely unchanged (Table 3).

**Table 2:** Procedure details of all patients

No. of patients	Length of stricture (cm) (Left/Right)	Size of SEMS (Diameter×Length) (Left/Right)	No. of ablations	Duration of ablation (s)	Ablation energy (watts)
1	6.4/8.1	6 mm × 8 cm/7 mm × 10 cm	2	100	10
2	4.5*	8 mm × 6 cm*	3	90	10
3	3.4/4.5	8 mm × 4 cm/8 mm × 4 cm	2	90	10
4	2.7*	8 mm × 4 cm*	2	90	10
5	4.7/5.0	8 mm × 6 cm/8 mm × 6 cm	2	120	6
6	5.1/6.2	8 mm × 6 cm/6 mm × 8 cm	2	90	10
7	5.7**	8 mm × 8 cm**	2	90	10
8	5.1*	8 mm × 8 cm*	2	90	10
9	6.0*	8 mm × 6 cm*	3	60	10

* : Right;

** : Left.

**Table 3:** Routine blood and biochemical level changes before and after procedure

Biochemical levels	Before treatment (mean ± SD)	After treatment (day 1–3) (mean ± SD)	Normal range
WBC (×10^9^/L)	10.98 ± 1.40	11.88 ± 1.12	4.00-10.00
Hb (g/L)	107.33 ± 7.46	102.67 ± 8.54	120–160
PLT (×10^9^/L)	268.22 ± 26.03	295.33 ± 35.84	100–300
TB (μmol/L)	137.11 ± 29.27	117.54 ± 31.66	3.0–22.0
DB (μmol/L)	87.88 ± 18.99	62.06 ± 17.21	0.5–7.0
ALB (g/L)	34.72 ± 1.83	32.38 ± 1.22	35.0–50.0
ALT (U/L)	110.11 ± 35.33	68.44 ± 15.31	1–40
AST (U/L)	72.78 ± 12.82	68.78 ± 16.66	1–37
ALP (U/L)	333.89 ± 48.86	323.44 ± 57.59	0–110
GGT (U/L)	431.22 ± 92.26	327.44 ± 73.52	2–50
LDH (U/L)	195.56 ± 10.25	198.22 ± 26.66	114–240
AMYL (U/L)	163.67 ± 47.36*	97.67 ± 23.42	30–110
LIPA (U/L)	920.56 ± 391.92*	264.67 ± 124.89	23–300
UREA (mmol/L)	5.77 ± 0.65	7.31 ± 1.39	2.9–8.6
CREA (μmol/L)	74.11 ± 6.33	75.11 ± 7.59	53–115

WBC: white blood cell: Hb: hemoglobin; PLT: platelet; TB: total bilirubin; DB: direct bilirubin; ALB: albumin; ALT: alanine aminotransferase; AST: aspartate aminotransferase; ALP: alkaline phosphatase, GGT: gamma-glutamyl transpeptidase; LDH: lactate dehydrogenase; AMYL: amylase; LIPA: lipase; UREA: blood urea nitrogen; CREA: serum creatinine.

* :Post-operative quick-check levels.

**Figure 1:**
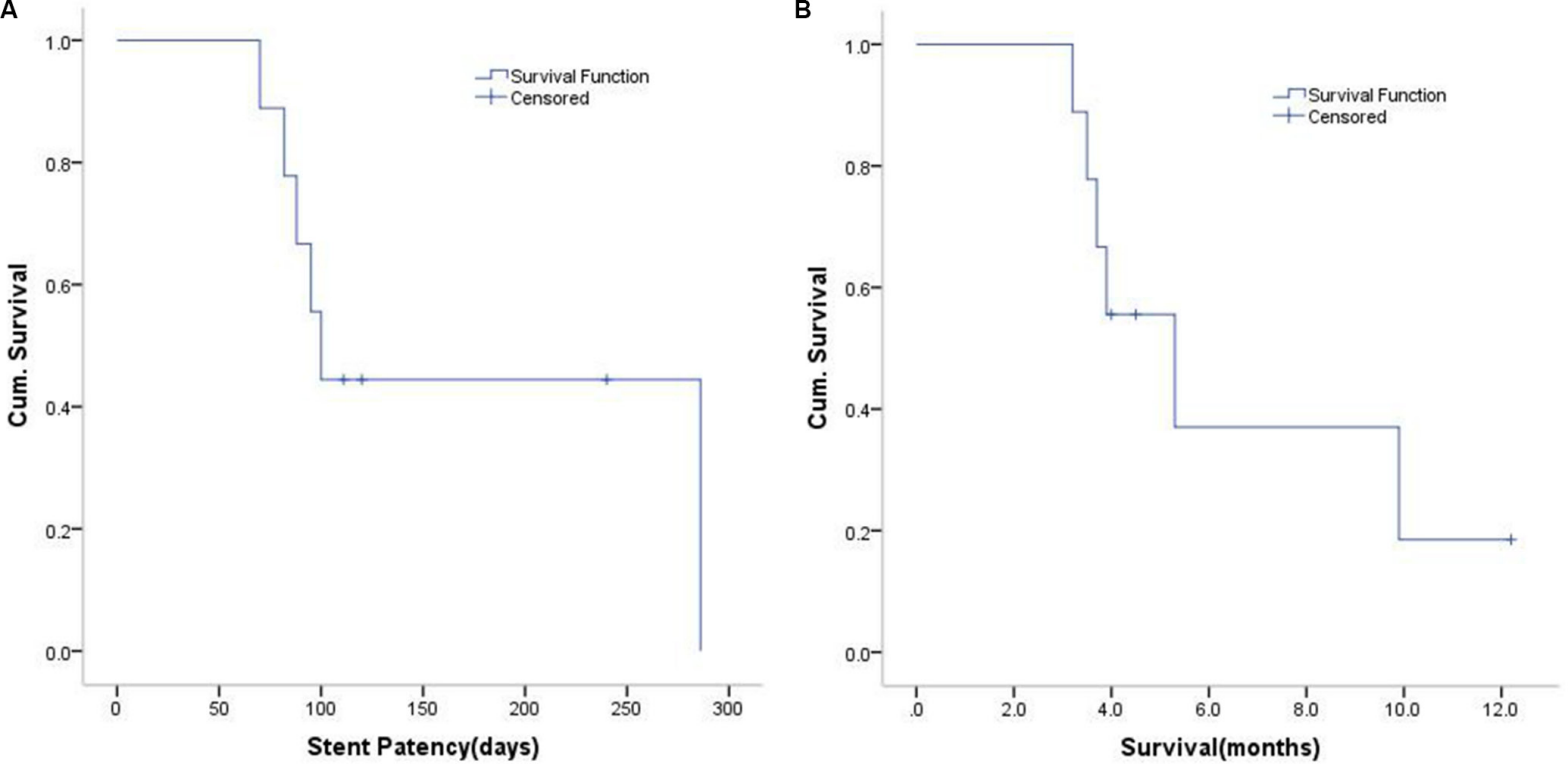
**(A)** Kaplan-Meier curve of stent patency. The calculation starts on the day of the first RFA procedure and extends to the time of proven stent occlusion, stent migration, or patient death. (**B**) Kaplan-Meier survival curve of the study patients. The calculation starts on the date of diagnosis and extends to the date of death.

The mean hospital stay was 18 ± 1.7 days (range, 8–24 days). There was no 30-day or hospital mortality. There were also no early major complications, such as bile duct perforation, bile leak, hemorrhage, or pancreatitis after the intervention. There was 3 patients suffered with pain (3 of 9) which could be alleviated with analgesics. There were 4 patients with cholangitis (4 of 9) cured with antibiotics. One patient developed recurrent cholangitis during the follow-up period; it was successfully managed with antibiotic therapy. However, one patient who had coronary heart disease with percutaneous coronary intervention, atrial fibrillation, hypertension, and hyperthyroidism presented with atrial fibrillation with a rapid ventricular rate and sudden onset of chronic heart failure after the RFA procedure; this was controlled with medical treatment. Two patients were also treated with chemotherapy (oxaliplatin plus tegafur gimeracil oteracil), and one ceased oxaliplatin during the first cycle because of unacceptable gastrointestinal toxicity. At the end of the follow-up period, three patients remained alive. One patient died of stroke, one of heart disease, and five of tumor progression. Median stent patency was 100 days (95% confidence interval (CI), 85–115 days). Overall median survival (from initial diagnosis until death) was 5. 3 months (95% CI, 2.5–8.1 months; Figure 1).

## DISCUSSION

Preoperative jaundice has been proven an independent risk factor for poor prognosis, and preoperative biliary drainage confers significant effects on reducing bilirubin levels [23]. Previous studies have shown that such techniques are not effective in reducing post-operative morbidity and mortality rates [24]. Although such procedures are thought to be associated with increased cholangitis and prolonged hospital stays [25], drainage should still be considered in patients at high-risk for cholangitis and liver failure from unrelieved obstruction jaundice [26]. Biliary decompression, usually biliary drainage using either an endoscopic (ERCP) or a percutaneous approach combined with biliary stent placement, has become the preferred method for palliative treatment of unresectable Hi CC. A key therapeutic factor in prolonging survival and improving quality of life is the prevention of post-operative biliary drainage complications [26]. However, long-term biliary drainage is underwhelming and modifications to stent design have not been proven effective in prolonging stent patency [27].

SEMS have been proven superior to plastic stents because of better durability, less frequent invasive procedures, and longer survival [28]. SEMS have become the standard treatment for patients with unresectable malignant biliary obstruction whose life expectancy is longer than 3 months [29, 30]. However, tumor growth through the uncovered stent mesh and epithelial hyperplasia, biofilm deposition, and sludge limit median metal stent patency to 6 months [27]. The stent patency rate is even lower in patients with Hi CC. Although some studies have reported that uncovered metal stent failure can potentially be corrected with covered SEMS, the effects of covered metal stents remain controversial. Covered stent use may cause dysfunction due to sludge formation and tumor overgrowth, thus increasing the incidence of cholecystitis and pancreatitis, which are associated with significant morbidity and mortality [26, 31, 32].

Given that standard biliary drainage results are underwhelming and stent patency rates for patients with Hi CC are low, patients in the present study were treated with percutaneous intraductal radiofrequency ablation combined with uncovered self-expand metal stent placement after percutaneous transhepatic cholangial drainage. In patients with malignant obstructive jaundice, endoscopic drainage has the advantages of less pain, absence of an uncomfortable external drainage tube, and lower risk of biliary peritonitis. However, in high level obstructions, bilateral or multiple strictures, endoscopic stent placement is difficult or impossible, with a lower success rate than the percutaneous route. Hence, a percutaneous route may be preferable in patients with Hi CC as opposed to endoscopic treatment [31, 33, 34].

Preliminary studies of RFA in animal experiments and clinical practice had very encouraging results. *Ex vivo* and *in vivo* porcine studies had confirmed the feasibility of the technique, providing preliminary safety data and defining appropriate power settings for human studies [35]. Endobiliary RFA application prior to SEMS placement has also been proven safe and effective in malignant biliary strictures. However, there were few reports on percutaneous approach of this technique for the treatment of malignant biliary obstruction [14, 31, 32, 36]. Malkhaz et al. recruited 39 patients with malignant biliary obstruction and demonstrated no early major complications or 30-day mortality, and only 38.5% of patients complained of postoperative pain. Their outcomes were favorable as compared to recently published large case studies on the treatment of malignant biliary obstruction through a percutaneous route without RFA [14, 37]. However, there were only 11 cases of Bismuth types III and IV Hi CC in Malkhaz's study, with a poor median survival of 89.5 days and median stent patency of 84.5 days. One recent study has reported that percutaneous transhepatic cholangiography and intraductal RFA combined with biliary stent placement for malignant biliary obstruction has similar 1-week jaundice remission and 3-month stent patency rates; however, the 6-month stent patency rate was higher than those of percutaneous transhepatic cholangiography or biliary stent placement alone [31]. This technique may have promising long-term benefits for patients; however, only five Hi CC cases were included in the cohort of 26 patients, and overall survival was not reported.

Our study showed that percutaneous intraductal RFA combined with SEMS placement should be safe and feasible in the treatment of unresectable Bismuth types III and IV Hi CC. All of our patients were diagnosed with Hi CC that originated from the bile duct epithelium and not cases involving the bile duct, such as is seen in liver or pancreatic cancers. We emphasize that the coagulation zone produced by a bipolar RF catheter is too small in patients who have tumors involving the bile duct. For most patients in our study, RFA of bilateral intrahepatic bile ducts was required. For patients with a long segmental obstruction of the common bile duct, RFA was performed section by section. In our study, one patient with a stent blockage 252 day post-procedure underwent repeat ablation; he was still alive at the end of the follow-up period. Re-ablation is easy to perform and minimally invasive, and can be also used to clear occluded metal stents [32]. One previous report asserted that ablation treatment should be performed cautiously because of excessive charring leading to perforation [31]. We agree that intraductal RFA can destroy the tumor tissue to some extent to enlarge the lumen for stent placement, leading to blood vessel loss; this is in agreement with the findings of Monga et al. [21]. Median stent patency and overall survival compare favorably with those of prior reports [14]. However, another retrospective study of 84 consecutive applications, endoscopic RFA for malignant biliary obstruction led to 17.9 months of median survival from the time of initial diagnosis and 170 days of median stent patency [38]. Because of the shortage of patient cases and larger prospective studies, whether an endoscopic approach is more effective and safe than the percutaneous route warrants further study [31].

Serious complications including bile duct perforation, bile leak, peribiliary sepsis, hemorrhage, or thermal injury to the duodenum or pancreas were not observed in our study, although they have been reported in previous studies using an endoscopic route. In addition, prior studies have reported other complications such as liver infraction, symptoms of cholangitis, abdominal pain, chills, and fever. Fortunately, each of these complications can be controlled with symptomatic and supportive treatment with low incidence, leading to few deaths [20, 31, 36, 39]. Although five cases of asymptomatic transient increase in serum amylase were observed, no cases of clinical pancreatitis were observed. We consider that these results may be associated with preoperative fasting, water, and prophylactic use of octreotide acetate. However, because of the small sample size, whether these techniques are preventative requires further study. In addition, four cases of procedure-related cholangitis were observed, which can be controlled with antibiotic agents. With a high retrograde infection rate, we recommend that antibiotics should be regularly used prophylactically in these patients. We speculate that cholangitis may be associated with the wire guide that was advanced into the duodenum, carrying intestinal bacteria that resulted in retrograde infection. Indeed, Li et al. have reported that prophylactic antibiotic agent administration in single doses may be necessary for these patients [31]. We also observed that atrial fibrillation and sudden onset of chronic heart failure were induced in one patient after the procedure, which may present a relative contraindication for this technique.

The limitations of our study should be noted. Foremost, this is a single-center study using retrospective methods and a non-randomized design. Therefore, selection bias was unavoidable and the results may not be fully generalizable, in particular to patients of other etiologies. Additionally, the sample size of our study is small. However, Hi CC is a rare disease and is thus difficult to study via large, randomized, single-center studies. In addition, long-term follow-up is required to determine whether this technique can prolong patient survival. Finally, our study lacks a control group; further studies using randomization to compare stent placement alone with RFA plus stent placement are required.

In conclusion, percutaneous intraductal RFA combined with metal stent placement is a feasible and safe treatment strategy in unresectable Bismuth Types III and IV Hi CC. A large multicenter prospective study, preferably a randomized control trial, is required to confirm the benefits of long-term stent patency and survival.

## MATERIALS AND METHODS

### Patient selection

**Figure 2:**
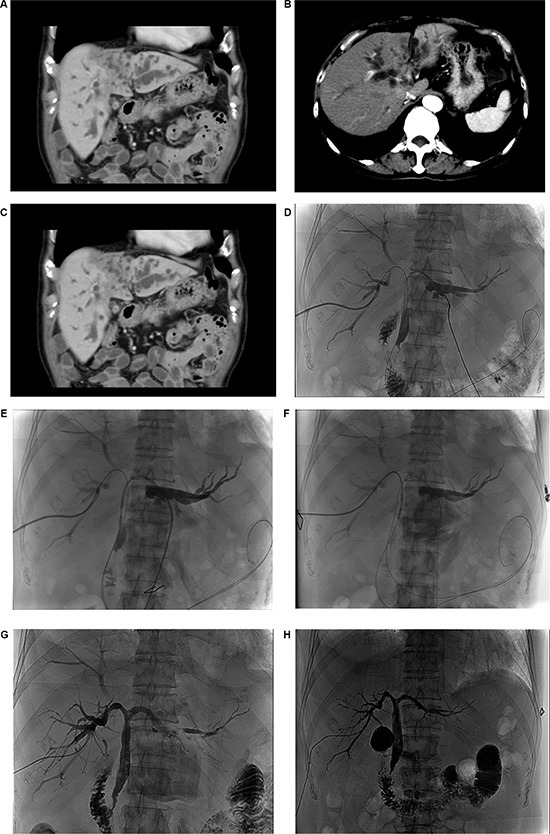
(**A**–**C**) Computed tomography scans show an inoperable Bismuth type IV hilar cholangiocarcinoma. (**D**) A percutaneous transhepatic cholangiography shows the stricture positioning. (**E** and **F**) Percutaneous radiofrequency ablation for the left and right hepatic duct. (**G**) Metal stent in position. (**H**) A cholangiogram showing stent patency four days after the RFA treatment.

The study was approved by the ethics committee of our hospital, and written informed consent was obtained from each patient in according with the Declaration of Helsinki. Eligible patients were ≥ 18 years of age with clinical symptoms at presentation, medical diagnoses confirmed as Bismuth types III and IV Hi CC (Figure 2A–2C), and an Eastern Cooperative Oncology Group (ECOG) performance status of 0–2. Patients were excluded for a history of a secondary malignancy, or if they were lost to follow-up, or had missing data.

### Treatment and assessments

All patients underwent ultrasound (US)-guided PTCD prior to the procedure. At the time of PTCD, hematological, biochemical, and coagulation tests were performed. Subsequently, preoperative fasting and water were prepared for approximately 6 to 8 hours, followed by percutaneous intraductal RFA combined with SEMS placement. On the first preoperative and postoperative day, octreotide acetate (0.1 mg) was administered by intramuscular injection three times a day to prevent acute pancreatitis. Three to four days later, with the guidance of digital subtraction angiography (DSA), the catheter was removed if the stent was draining following recheck cholangiography (Figure 2H).

### RFA procedure

**Figure 3:**
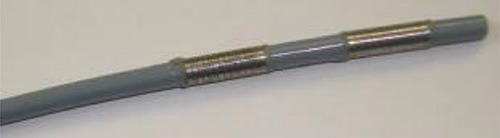
The Habib™ EndoHBP is a bipolar RF catheter with two radiologically marked electrodes at its tip

The Habib™ EndoHBP is an 8 Fr. (2.6 mm), 1.8 m long bipolar RF catheter with two radiologically marked electrodes at its tip that inserts over a 0.035 inch guide wire into the bile duct (Figure 3). This catheter can be used either for an endoscopic retrograde cholangiopancreatography (ERCP) or a percutaneous transhepatic cholangiography (PTC) procedure. Under digital subtraction angiography (DSA) guidance, PTC was performed to locate the biliary obstruction and to confirm its length and diameter. A guide wire was then passed through the stenosis via the PTCD catheter (Figure 2D). The tip of the probe was then advanced over the wire, with the tip of the probe placed across the malignant stricture. It was then attached to a standard high-frequency generator, and 6–10 W are typically applied for 90 to 120 s followed immediately by SEMS placement (Figure 2E and 2F). Depending on the stricture size, the RFA energy and time can be controlled at different tumor sites during one procedure. Immediately after RFA, an uncovered SEMS (Wallstent; Boston Scientific, Boston, Mass) mounted on a delivery system was placed (Figure 2G).

The primary endpoint was the technical safety and feasibility of percutaneous intraductal RFA. Secondary outcomes were overall survival, stent patency, and complications. Stent patency was counted from the time of the first electively performed RFA procedure to the time of proven stent occlusion, stent migration, or patient death. Overall survival was calculated from the date of diagnosis until the date of death. Patients who died were excluded at the date of their last follow-up.

### Statistical analysis

Descriptive statistics are shown as mean ± standard deviation (SD) or median and range, as appropriate. Stent patency and overall survival were analyzed with Kaplan-Meier curves. All analyses were performed with SPSS statistics software (Version 19.0). The database was closed for analysis in May 2015.
